# Does the Subject Content of the Pharmacy Degree Course Influence the Community Pharmacist’s Views on Competencies for Practice?

**DOI:** 10.3390/pharmacy3030137

**Published:** 2015-09-01

**Authors:** Jeffrey Atkinson, Kristien De Paepe, Antonio Sánchez Pozo, Dimitrios Rekkas, Daisy Volmer, Jouni Hirvonen, Borut Bozic, Agnieska Skowron, Constantin Mircioiu, Annie Marcincal, Andries Koster, Keith Wilson, Chris van Schravendijk, Jamie Wilkinson

**Affiliations:** 1 Pharmacology Department, Lorraine University, 5 rue Albert Lebrun, 54000 Nancy, France; 2 Pharmacolor Consultants Nancy, 12 rue de Versigny, Villers 54600, France; 3 Department of Pharmacy, Vrije Universiteit Brussel, Laarbeeklaan 103, Brussels 1090, Belgium; E-Mail: kdepaepe@vub.ac.be; 4 Faculty of Pharmacy, University of Granada (UGR), Campus Universitario de la Cartuja s/n, Granada 18701, Spain; E-Mail: sanchezp@ugr.es; 5 School of Pharmacy, National and Kapodistrian University Athens, Panepistimiou 30, Athens 10679, Greece; E-Mail: rekkas@pharm.uoa.gr; 6 Pharmacy Faculty, University of Tartu, Nooruse 1, Tartu 50411, Estonia; E-Mail: daisy.volmer@ut.ee; 7 Pharmacy Faculty, University of Helsinki, Yliopistonkatu 4, P.O. Box 33-4, Helsinki 00014, Finland; E-Mail: jouni.hirvonen@helsinki.fi; 8 Faculty of Pharmacy, University of Ljubljana, Askerceva cesta 7, Ljubljana 1000, Slovenia; E-Mail: Borut.Bozic@ffa.uni-lj.si; 9 Pharmacy Faculty, Jagiellonian University, UL, Golebia 24, Krakow 31-007, Poland; E-Mail: askowron@cm-uj.krakow.pl; 10 Pharmacy Faculty, University of Medicine and Pharmacy “Carol Davila” Bucharest, Dionisie Lupu 37, Bucharest 020021, Romania; E-Mail: constantin.mircioiu@yahoo.com; 11 European Association of Faculties of Pharmacy, Faculty of Pharmacy, Université de Lille 2, Lille 59000, France; E-Mail: annie.marcincal@pharma.univ-lille2.fr; 12 European Association of Faculties of Pharmacy, Department Pharmaceutical Sciences, Utrecht University, PO Box 80082, 3508 TB Utrecht, The Netherlands; E-Mail: A.S.Koster@uu.nl; 13 School of Life and Health Sciences, Aston University, Birmingham, B4 7ET, UK; E-Mail: k.a.wilson@aston.ac.uk; 14 Vrije Universiteit Brussel, Laarbeeklaan 103, 1090 Brussels, Belgium; E-Mail: chrisvs@vub.ac.be; 15 Pharmaceutical Group of the European Union (PGEU), Rue du Luxembourg 19, 1000 Brussels, Belgium; E-Mail: j.wilkinson@pgeu.eu

**Keywords:** pharmacy, education, community, practice, competence

## Abstract

Do community pharmacists coming from different educational backgrounds rank the importance of competences for practice differently—or is the way in which they see their profession more influenced by practice than university education? A survey was carried out on 68 competences for pharmacy practice in seven countries with different pharmacy education systems in terms of the relative importance of the subject areas chemical and medicinal sciences. Community pharmacists were asked to rank the competences in terms of relative importance for practice; competences were divided into personal and patient-care competences. The ranking was very similar in the seven countries suggesting that evaluation of competences for practice is based more on professional experience than on prior university education. There were some differences for instance in research-related competences and these may be influenced, by education.

## 1. Introduction

In 1985, the then European Economic Community (EEC) published a directive [1] on pharmacy practice that assumed that pharmacy education in the EEC was broadly comparable and, thus, that the European education system was producing pharmacists with similar competences. In the early 1990s, the European Association of Faculties of Pharmacy (EAFP) [2] questioned these assumptions [3]. EAFP surveyed pharmacy courses in the 11 EEC members and found that although the emphasis in most faculties was on chemical sciences, there was great variability in pharmacy degree courses in the EEC regarding the percentages of time spent on different subjects [4].

At that time it was hoped that European integration would produce greater harmonization in pharmacy education and therefore in competences for practice. In 2011, the PHARMINE (“*PHARMacy Education IN Europe”*) project [5] revisited this problem. In the 20-year interval between the two studies there was a shift in several countries from chemical to medicinal sciences, albeit, overall variability in degree courses from country to country had not decreased [6]. PHARMINE reflected upon whether differences in pharmacy degrees could be minored by expressing content as competences rather than subjects.

As a follow-up to PHARMINE, a second study, the PHAR-QA (“*Quality Assurance in European PHARmacy-Education and Training*”) project [7], again funded by the European Commission, asked community pharmacists to rank competences for pharmacy practice.

This paper combines the results of the PHARMINE and PHAR-QA studies. It looks at whether the nature of the degree course (in terms of the relative importance of the subject areas chemical and medicinal sciences taken as an indication of a more “scientific” or a more “clinical” course) has any influence on the way in which community pharmacists ranked the competences they consider are required for practice. The paper evaluates to what extent university education or professional experience can influence the way in which practicing community pharmacists judge their *métier* and how the balance between these two factors could be altered by the introduction of competence-based education.

## 2. Experimental Section

In the PHARMINE project, country profiles for pharmacy education and training were drawn up with the help of academics, students, professional pharmacists, and their organizations, as well as representatives of different governmental bodies concerned with pharmacy in the 47 countries of the European Higher Education Area [8]. Amongst others, one of the areas explored in the country profiles was the structure of the pharmacy degree course that was divided into six subject areas: chemical sciences, medicinal sciences, biological sciences, pharmaceutical technology, and law and societal issues. A subject area course index was calculated as: ((percentage of contact hours spent on medicinal subjects/percentage of contact hours spent on chemical subjects) x 100) using data from the PHARMINE study, as given in the 2014 paper on heterogeneity of pharmacy education cited above [6].

In the PHARMINE study, “medicinal subjects” included contact hours in the subjects of anatomy, physiology, medical terminology, pathology, histology, nutrition, pharmacology/pharmacotherapy, toxicology, hematology, immunology, parasitology, hygiene, emergency therapy, non-pharmacological treatment, clinical chemistry/bio-analysis, radiochemistry, dispensing process, drug prescription, prescription analysis (detection of adverse effects and drug interactions), generic drugs, planning, running and interpretation of the data, of clinical trials, medical devices, orthopedics, over the counter medicines, complementary therapy, at-home support and care, skin illness and treatment, homeopathy, phyto-therapy, drugs in veterinary medicine, pharmaceutical care, pharmaceutical therapy of illness, and disease. “Chemical subjects” included contact hours in the subjects of general and inorganic chemistry, medical physico-chemistry, organic chemistry, pharmacopeia analysis, analytical chemistry, and pharmaceutical chemistry including analysis of medicinal products.

Ranking data on competences for practice were taken from the PHAR-QA *surveymonkey* [9] questionnaire that was available online from 14 January 2014 to 1 November 2014 *i.e.*, 8.5 months. Contacts were made by electronic and other means with the same groups as in the PHARMINE study (see previously). *Post hoc* analysis of the data allowed the creation of six subgroups: academics, students, community pharmacists, hospital pharmacists, industrial pharmacists, and pharmacists working in other areas. Here we will present the data for community pharmacists; data for other professional categories will be presented elsewhere [10].

The first six questions of the PHAR-QA survey were on the profile of the respondent asking, amongst others, country of residence, current occupation, and duration of activity.

Questions seven through 19 asked about 13 groups of competences with a total of 68 competences (see annex). Questions in groups seven through 11 were concerned with personal competences and in groups 12 through 19 with patient care competences.

Respondents were asked to rank the proposals for competences with a Likert scale:

1. Not important = Can be ignored.
2. Quite important = Valuable but not obligatory.
3. Very important = Obligatory with exceptions depending upon field of pharmacy practice.
4. Essential = Obligatory.

Results are presented in the form of “scores” based on the methodology used in MEDINE2 [11]: score = (frequency rank 3 + frequency rank 4) as % of total frequency. Scores give more granularity and a better pictorial representation than the basic Likert data. Data were obtained from 39 European countries. Data presented here are from the seven European Union member states in which the number of respondents was > 10 (Table 1). Analysis was limited to the European Union as its 28 member states come under the directive on sectoral professions such as pharmacy [12]. One of the annexes of this directive lists the subject areas that are to be taught in the pharmacy degree course in the European Union. Of the 28 member states only seven provided 10 or more community pharmacists respondents.

### Statistical Analysis

Results are also expressed as medians with 25 and 75% percentiles; differences among countries were analyzed using the Kruskal-Wallis test followed by Dunn’s multiple comparisons test. All statistics were performed using GraphPad software [13].

## 3. Results and Discussion

In Table 1 are the medians for duration of practice. Kruskal-Wallace analysis showed a significant effect of country (*P* = 0.0014) and the Dunn’s multiple comparisons test showed that the duration of practice of the respondents from the Czech Republic was lower than that of respondents from Germany, Ireland or Spain. None of the other comparisons were significant.

Table 1 also shows the medicinal sciences/chemical sciences scores. In Germany the degree course is more “chemical”; in Belgium, the Czech Republic, and Spain the importance of the two subject areas is equal; in The Netherlands and the United Kingdom there is a more “medicinal” course, and this is even more pronounced in Ireland. The medicinal/chemical ratio varies almost four-fold from Germany (0.7) to Ireland (2.6).

Finally, Table 1 shows overall the median rankings for competences (*n* = 68). The Kruskal-Wallis test showed a significant difference amongst countries (*P* = 0.0006) with a significantly higher median for Spain compared to Belgium, Germany, and Ireland. None of the other multiple comparisons amongst countries reached statistical significance.

**Table 1:** Characteristics of the seven countries, the medicinal sciences/chemical sciences indices (latter data from the PHARMINE study), and the rankings for competences.

Country	Number of respondents	Duration of activity (years; median, 25% and 75% percentiles)	Medicinal sciences %	Chemical sciences %	Medicinal/chemical score	Ranking of competences (median, 25% and 75% percentiles, *n* = 68)
Belgium	25	10/5/20	24	27	1.1	81/63/91
Czech Republic	15	5/5/15	19	17	1.1	84/67/92
Germany	13	30/15/30	28	40	0.7	82/67/92
Ireland	13	20/10/33	36	14	2.6	77/55/92
Spain	27	15/10/30	28	24	1.2	91/82/96
The Netherlands	18	20/5/23	31	20	1.6	82/57/94
United Kingdom	48	10/5/20	24	14	1.7	87/59/96

**Figure 1:**
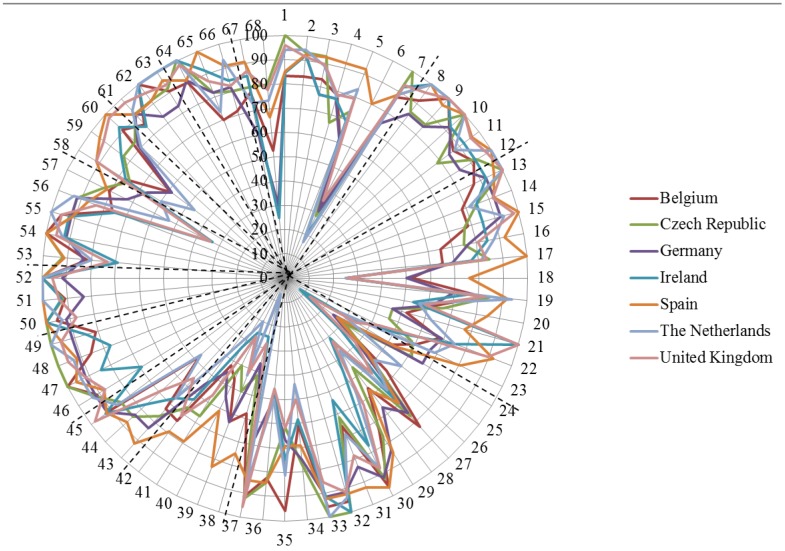
Radar chart of the ranking scores (on the central vertical axe, 0%–100%) for the 68 competences (on the circumference) by the seven countries (in different colours). Dotted lines separate the 13 competence groups (see annex) in Figure 1 are given the ranking scores for the 68 competences by the seven countries.

Figure 1 shows the ranking by the seven countries of the 68 competences. This is presented as a radar chart. Radar charts are a useful way to display multivariate observations with an arbitrary number of variables. It allows one to find clusters and also to identify outliers [14]. This radar chart presentation allows an easy overview of the global rankings of competences. It underlines the fact that overall the global rankings by the different countries are similar, with similar highs and lows. This is especially true for the left-hand side of the Figure that represents the rankings for the patient care competences (number 43 through 68). Opinions of the relative importance of such competences appear to be formed by work experience rather than university education. In answer to the question “do community pharmacists coming from different educational backgrounds rank the importance of competences for practice differently” the answer would be no in the case of patient care competences. Examination of Figure 1 shows that the ranking of competences for practice is very similar in seven countries that have different systems of pharmacy education. It should be noted that the ranking score is based on a combination of ranks 3 and 4 that specify that competences are “obligatory”.

There are more differences in the right-hand side of the Figure that represents personal competences. Competence 6 is an interesting case. The difference between minimum and maximum for country rankings in group 7 (“personal competences: learning and knowledge”) competence 6 (“ability to design and conduct research using appropriate methodology”) was large (63, see Table A1); Spain, which has a “balanced” course with a medicinal sciences/chemical sciences index of 1.2, ranked highest with 80%. Ireland and the Netherlands which have more “medical” indices of 2.6 and 1.6, respectively, showed the lowest rankings for competence 6: 18 and 17%, respectively. Spain also ranked highest for all competences. In the research-related group 11 (“personal competences: understanding of industrial pharmacy”) Spain scored highest for all 5 competences. Thus differences for Spain may be influenced by education rather than professional experience, albeit, Germany, which has a more “chemical” index (0.7), did not rank competence 6 or the competences in group 11 particularly high.

Several provisos should be added. It is possible that differences in ranking scores are related to duration of practice (*i.e.*, numbers of years since leaving university) rather than to course content. With the median years of practice being significantly different it could very well be that older pharmacists in a given country took a very different course of study 30–40 years ago than younger pharmacists from the same country. Furthermore, it is likely the mix of medicinal/chemical subjects would have differed within countries for participants dependent on when they studied especially as there has been a move towards more medicinal sciences in the past 20 years (see introduction). This cannot be tested, however, as numbers in the different groups do not allow the creation of subgroups based on duration of practice. Some comments can be made on the basis of the existing data. The community pharmacist respondents from the Czech Republic were younger than in several other countries, but the Czech Republic community pharmacists did not show any marked differences with other countries as far as ranking of competences was concerned. Spanish community pharmacists did show a specific pattern of ranking in several groups of competences but their median duration of practice was mid-range.

The conclusion of this paper relies on the fact that the curricula investigated are as different as possible in the relative importance of “medicinal” versus “chemical” sciences component. The seven countries selected were selected on the basis of providing more than 10 respondents. Nonetheless they do represent a significantly wide range of scores. Ireland has the highest value of the 26 European Union member states that have pharmacy departments (1^st^/26), and Germany the 3rd from the lowest (23^rd^/26)^vi^.

The PHARMINE study cited above showed that a competence approach is rarely used in pre-graduate pharmacy education in Europe. There have been several studies on the use of a competence framework to monitor and improve pharmacy practice in a working environment. A study using the general level framework with Singaporean hospital pharmacists showed that all but eight of the 63 behavioral descriptors improved in nine months [15]. A similar study with hospital pharmacists in Queensland showed an improvement in 35 out of 61 competences [16]. Studies have also been conducted in Canada [17]. and elsewhere. The results of all these studies are that competence frameworks are useful tools to monitor and improve performance.

## 4. Conclusions

This study shows that community pharmacists largely form their opinions on the importance of competences of the basis of work experience rather than university education. The move to harmonize European pharmacy practice expressed in the 1980s seems to have been successful, as judged from the similar way in which community pharmacists from different countries rank competences for practice. However this is less the result of harmonization of pharmacy education that still shows wide diversity.

The short-term perspective of this work is the modification of the existing questionnaire according to the results obtained and the endorsement of the modified version.

The long-term perspective is the introduction of competence-based learning into the university curriculum for pharmacy. This is being discussed in Australia and New Zealand [18] and elsewhere. It now needs to be considered in Europe. Our results suggest that differences in university pharmacy programs are not crucial in the development of specific competencies (at least in the field of community pharmacy, where the majority of pharmacists work). Thus, we do not need a very stringent and tight framework for curricula of pharmacy education. Academia provides graduates with competencies as “novices” (according to five-stage model of competencies proposed by Dreyfus and Dreyfus, 1980 [19]). Thus, competence-based learning in universities would provide a sound foundation allowing graduates to gather experience through practical training in the real job environment. Furthermore, academic freedom as to course content should be incorporated into quality assurance of pharmacy education especially when EU directive is “translated” into national frameworks.

## Appendix

**Table A1:** Ranking of competences by countries.

Seq.	Competence	Belgium	Czech Republic	Germany	Ireland	Spain	The Netherlands	United Kingdom	Min.	Max.	Range: max–min.
	Group 7. Personal competences: learning and knowledge										
1	1. Ability to identify learning needs and to learn independently (including continuous professional development (CPD)).	83	100	85	85	85	94	96	83	100	17
2	2. Analysis: ability to apply logic to problem solving, evaluating pros and cons and following up on the solution found.	83	93	92	92	93	94	92	83	94	11
3	3. Synthesis: capacity to gather and critically appraise relevant knowledge and to summarise the key points.	83	93	77	77	93	89	90	77	93	16
4	4. Capacity to evaluate scientific data in line with current scientific and technological knowledge.	78	67	77	77	92	78	79	67	92	25
5	5. Ability to interpret preclinical and clinical evidence-based medical science and apply the knowledge to pharmaceutical practice.	63	73	62	69	92	83	79	62	92	31
6	6. Ability to design and conduct research using appropriate methodology.	33	29	31	18	80	17	36	17	80	63
7	7. Ability to maintain current knowledge of relevant legislation and codes of pharmacy practice.	88	100	75	92	89	89	93	75	100	25
	Group 8. Personal competences: values										
8	1. Demonstrate a professional approach to tasks and human relations.	92	86	85	100	100	100	96	85	100	15
9	2. Demonstrate the ability to maintain confidentiality.	100	86	85	100	96	100	100	85	100	15
10	3. Take full personal responsibility for patient care and other aspects of one’s practice.	92	100	92	92	100	100	100	92	100	8
11	4. Inspire the confidence of others in one’s actions and advice.	87	79	85	92	96	88	96	79	96	18
12	5. Demonstrate high ethical standards.	92	93	85	92	100	100	98	85	100	15
	Group 9. Personal competences: communication and organisational skills.										
13	1. Effective communication skills (both orally and written).	87	100	92	92	96	100	100	87	100	13
14	2. Effective use of information technology.	78	85	92	85	92	81	92	78	92	14
15	3. Ability to work effectively as part of a team.	78	77	92	85	100	94	98	77	100	23
16	4. Ability to identify and implement legal and professional requirements relating to employment (e.g., for pharmacy technicians) and to safety in the workplace.	65	75	77	85	92	88	81	65	92	27
17	5. Ability to contribute to the learning and training of staff.	65	85	69	77	100	81	83	65	100	35
18	6. Ability to design and manage the development processes in the production of medicines.	52	25	50	25	76	25	26	25	76	51
19	7. Ability to identify and manage risk and quality of service issues.	82	85	69	75	92	94	83	69	94	25
20	8. Ability to identify the need for new services.	62	67	62	54	85	56	59	54	85	31
21	9. Ability to communicate in English and/or locally relevant languages.	52	46	46	100	77	63	100	46	100	54
22	10. Ability to evaluate issues related to quality of service.	68	46	69	75	92	75	90	46	92	46
23	11. Ability to negotiate, understand a business environment and develop entrepreneurship.	61	58	67	50	81	69	43	43	81	37
	Group 10. Personal competences: knowledge of different areas of the science of medicines.										
24	1. Plant and animal biology.	52	62	67	31	54	27	35	27	67	40
25	2. Physics.	26	31	25	8	27	60	13	8	60	52
26	3. General and inorganic chemistry.	57	46	42	31	46	50	39	31	57	26
27	4. Organic and medicinal/pharmaceutical chemistry.	83	77	75	69	69	63	53	53	83	29
28	5. Analytical chemistry.	57	46	67	31	58	38	33	31	67	36
29	6. General and applied biochemistry (medicinal and clinical).	74	77	83	46	85	56	62	46	85	38
30	7. Anatomy and physiology; medical terminology.	96	92	92	77	96	88	88	77	96	19
31	8. Microbiology.	65	62	83	54	92	69	78	54	92	38
32	9. Pharmacology including pharmacokinetics.	96	100	92	100	92	94	91	91	100	9
33	10. Pharmacotherapy and pharmaco-epidemiology.	96	100	92	92	92	100	85	85	100	15
34	11. Pharmaceutical technology including analyses of medicinal products.	61	77	75	58	69	44	50	44	77	33
35	12. Toxicology.	96	62	67	75	69	81	62	62	96	34
36	13. Pharmacognosy.	83	85	50	46	85	50	46	46	85	39
37	14. Legislation and professional ethics.	91	92	67	92	85	88	96	67	96	29
	Group 11. Personal competences: understanding of industrial pharmacy.										
38	1. Current knowledge of design, synthesis, isolation, characterisation and biological evaluation of active substances.	58	42	36	25	75	7	28	7	75	68
39	2. Current knowledge of good manufacturing practice (GMP) and of good laboratory practice (GLP).	63	50	64	25	83	43	42	25	83	58
40	3. Current knowledge of European directives on qualified persons (QPs).	47	40	55	25	61	20	27	20	61	41
41	4. Current knowledge of drug registration, licensing and marketing.	42	67	45	33	79	27	57	27	79	53
42	5. Current knowledge of good clinical practice (GCP).	74	67	55	63	79	40	71	40	79	39
	Group 12. Patient care competences: patient consultation and assessment.										
43	1. Ability to perform and interpret medical laboratory tests.	73	77	83	67	92	67	56	56	92	36
44	2. Ability to perform appropriate diagnostic or physiological tests to inform clinical decision making e.g., measurement of blood pressure.	48	85	83	77	88	47	69	47	88	41
45	3. Ability to recognise when referral to another member of the healthcare team is needed because a potential clinical problem is identified (pharmaceutical, medical, psychological or social).	91	85	92	92	92	87	98	85	98	13
	Group 13. Patient care competences: need for drug treatment.										
46	1. Retrieval and interpretation of relevant information on the patient's clinical background.	91	92	92	69	88	93	87	69	93	24
47	2. Retrieval and interpretation of an accurate and comprehensive drug history if and when required.	100	100	92	85	96	93	91	85	100	15
48	3. Identification of non-adherence and implementation of appropriate patient intervention.	86	100	91	77	92	93	96	77	100	23
49	4. Ability to advise to physicians and—in some cases—prescribe medication.	81	100	91	85	96	100	96	81	100	19
	Group 14. Patient care competences: drug interactions.										
50	1. Identification, understanding and prioritisation of drug-drug interactions at a molecular level (e.g., use of codeine with paracetamol).	95	100	92	100	100	93	87	87	100	13
51	2. Identification, understanding, and prioritisation of drug-patient interactions, including those that preclude or require the use of a specific drug (e.g., trastuzumab for treatment of breast cancer in women with HER2 overexpression).	91	92	83	92	100	100	93	83	100	17
52	3. Identification, understanding, and prioritisation of drug-disease interactions (e.g., NSAIDs in heart failure).	100	100	92	100	100	100	96	92	100	8
	Group 15. Patient care competences: provision of drug product.										
53	1. Familiarity with the bio-pharmaceutical, pharmacodynamic and pharmacokinetic activity of a substance in the body.	82	92	83	69	91	80	73	69	92	22
54	2. Supply of appropriate medicines taking into account dose, correct formulation, concentration, administration route and timing.	100	100	92	92	100	93	96	92	100	8
55	3. Critical evaluation of the prescription to ensure that it is clinically appropriate and legal.	95	92	92	92	91	100	96	91	100	9
56	4. Familiarity with the supply chain of medicines and the ability to ensure timely flow of drug products to the patient.	76	92	92	75	87	93	83	75	93	18
57	5. Ability to manufacture medicinal products that are not commercially available.	81	83	73	33	82	53	34	33	83	50
	Group 16. Patient care competences: patient education.										
58	1. Promotion of public health in collaboration with other actors in the healthcare system.	77	75	67	77	91	60	91	60	91	31
59	2. Provision of appropriate lifestyle advice on smoking, obesity, *etc*.	59	83	58	85	96	47	93	47	96	49
60	3. Provision of appropriate advice on resistance to antibiotics and similar public health issues.	90	83	82	92	100	80	98	80	100	20
	Group 17. Patient care competences: provision of information and service.										
61	1. Ability to use effective consultations to identify the patient's need for information.	86	92	92	85	91	93	98	85	98	13
62	2. Provision of accurate and appropriate information on prescription medicines.	100	92	83	100	91	100	95	83	100	17
63	3. Provision of informed support for patients in selection and use of non-prescription medicines for minor ailments (e.g., cough remedies...)	90	92	83	100	96	100	93	83	100	17
	Group 18. Patient care competences: monitoring of drug therapy.										
64	1. Identification and prioritisation of problems in the management of medicines in a timely manner and with sufficient efficacy to ensure patient safety.	90	100	91	100	91	100	98	90	100	10
65	2. Ability to monitor and report to all concerned in a timely manner, and in accordance with current regulatory guidelines on Good Pharmacovigilance Practices (GVPs), Adverse Drug Events and Reactions (ADEs and ADRs).	70	82	82	92	100	73	87	70	100	30
66	3. Undertaking of a critical evaluation of prescribed medicines to confirm that current clinical guidelines are appropriately applied.	71	80	82	85	91	93	82	71	93	22
	Group 19. Patient care competences: evaluation of outcomes.										
67	1. Assessment of outcomes on the monitoring of patient care and follow-up interventions.	78	80	60	85	90	73	87	60	90	30
68	2. Evaluation of cost effectiveness of treatment.	53	80	30	25	67	73	78	25	80	55

Seq.: sequential numbering (as in Figure 1); Min.: minimum; Max.: maximum; Note that the numbering of the groups of competences starts at 7, *i.e.*, after the 6 questions on profile.
